# A framework for application of metabolic modeling in yeast to predict the effects of nsSNV in human orthologs

**DOI:** 10.1186/1745-6150-9-9

**Published:** 2014-06-03

**Authors:** Hayley Dingerdissen, Daniel S Weaver, Peter D Karp, Yang Pan, Vahan Simonyan, Raja Mazumder

**Affiliations:** 1 Department of Biochemistry and Molecular Biology, The George Washington University Medical Center, Ross Hall, Room 540, 2300 Eye Street NW, Washington, DC 20037, USA; 2 Bioinformatics Research Group, Artificial Intelligence Center, SRI International Menlo Park, Menlo Park, CA 94025, USA; 3 Center for Biologics Evaluation and Research, US Food and Drug Administration, 1451 Rockville Pike, Rockville, MD 20852, USA; 4 McCormick Genomic and Proteomic Center, George Washington University, Washington, DC 20037, USA

**Keywords:** nsSNV, Ortholog, Sequence conservation, FBA, Yeast metabolic modeling

## Abstract

**Background:**

We have previously suggested a method for proteome wide analysis of variation at functional residues wherein we identified the set of all human genes with nonsynonymous single nucleotide variation (nsSNV) in the active site residue of the corresponding proteins. 34 of these proteins were shown to have a 1:1:1 enzyme:pathway:reaction relationship, making these proteins ideal candidates for laboratory validation through creation and observation of specific yeast active site knock-outs and downstream targeted metabolomics experiments. Here we present the next step in the workflow toward using yeast metabolic modeling to predict human metabolic behavior resulting from nsSNV.

**Results:**

For the previously identified candidate proteins, we used the reciprocal best BLAST hits method followed by manual alignment and pathway comparison to identify 6 human proteins with yeast orthologs which were suitable for flux balance analysis (FBA). 5 of these proteins are known to be associated with diseases, including ribose 5-phosphate isomerase deficiency, myopathy with lactic acidosis and sideroblastic anaemia, anemia due to disorders of glutathione metabolism, and two porphyrias, and we suspect the sixth enzyme to have disease associations which are not yet classified or understood based on the work described herein.

**Conclusions:**

Preliminary findings using the Yeast 7.0 FBA model show lack of growth for only one enzyme, but augmentation of the Yeast 7.0 biomass function to better simulate knockout of certain genes suggested physiological relevance of variations in three additional proteins. Thus, we suggest the following four proteins for laboratory validation: delta-aminolevulinic acid dehydratase, ferrochelatase, ribose-5 phosphate isomerase and mitochondrial tyrosyl-tRNA synthetase. This study indicates that the predictive ability of this method will improve as more advanced, comprehensive models are developed. Moreover, these findings will be useful in the development of simple downstream biochemical or mass-spectrometric assays to corroborate these predictions and detect presence of certain known nsSNVs with deleterious outcomes. Results may also be useful in predicting as yet unknown outcomes of active site nsSNVs for enzymes that are not yet well classified or annotated.

**Reviewers:**

This article was reviewed by Daniel Haft and Igor B. Rogozin.

## Background

An enyzme’s active site specificity is an important determinant of functional catalysis. For this reason, if a nonsynonymous single nucleotide variation (nsSNV) occurs at the active site, thereby changing an active site amino acid residue, it is highly likely that enzyme activity will be lost or altered [1-7]. It follows that pathway activity should also be affected, with a potential end result of disease [8-10] or lethality.

In our previous paper [11] we used the SNVDis Tool [12], part of the High-performance Integrated Virtual Environment (HIVE) tools suite (accessed at hive.biochemistry.gwu.edu) [13] to identify the entire set of 559 human proteins with nonsynonymous variation at an active site residue resulting from a single nucleotide variation. Pathway, substrate and product annotation was manually retrieved from Kyoto Encyclopedia of Genes and Genomes (KEGG) [14] for all possible proteins in the dataset. To the best of our knowledge, a subset of 34 of the original proteins had a 1 enzyme:1 pathway:1 substrate/product set ratio, meaning these enzymes catalyze a single reaction for a single set of substrates and products and are currently annotated to participate in a single pathway. While there may be possible interaction between specified metabolites and alternative enzymes, the 1:1:1 relationship ensures no alternative metabolic interaction with the specified enzymes, thus making them suitable candidates for *in vivo* laboratory validation.

For this paper, we used the reciprocal best BLAST hits method [15] to query the set of 559 human active site nsSNV proteins against the yeast (*Saccharomyces cerevisiae*) proteome and, conversely, the top yeast protein hits back against the human proteome. We then checked for active site residue conservation and pathway/substrate/enzyme conservation between the 113 identified human-yeast orthologous pairs. We found 6 proteins which satisfied all criteria, 5 of which had human disease associations including anemias, porphyrias and others. The high incidence of dysfunction annotated among these active-site-nsSNV-containing proteins reinforces the notion that mutations in the active site disrupt normal enzyme and/or pathway activity. Although it may seem intuitive that the active site modifications are to blame for these disease associations, it is surprising to note that the disease-related annotations are not currently attributed to the active site variations for the 5 proteins in question, but are assigned to other sequence variations or causes.

Merely studying genomic variation may not be enough to determine the effects of active site variation, however, as one needs to examine the interactions and physiological outcome within the context of the cell, organ, or entire system [16]. Metabolic modeling is our attempt to test the impact of active site variation in a more meaningful way as such methods get more sophisticated. To this end, we used the Yeast 7.0 flux balance analysis (FBA) model [17] to predict the effects of deleting these 6 proteins, constructed with the relevant nsSNV active site mutation, on the growth of yeast. Since several of these proteins are involved in production of important metabolites that are not contained within the Yeast 7.0 biomass function, we repeated the analysis using a revised biomass equation that incorporates the relevant metabolites. FBA is an established mathematical and computational approach for studying metabolic networks [18,19]. FBA modeling has a number of biotech applications as it allows programmatic prediction of a metabolic phenotype using *in silico* computations of reaction stoichiometry without using reaction kinetics [20-22]. FBA results have been shown to correlate well with experimental observations [23].

In the future, predictions given by this *in silico* cell model can be subjected to laboratory validation using yeast cultures with site-induced mutagenesis to alter the proper active site residue and observe the outcomes with respect to growth rate, substrate and product quantities, byproduct generation and general viability. We reason that the conservation of these protein sequences from yeast to humans, in conjunction with conserved active site residues and conserved pathway interplay, provides strong evidence that similar outcomes are likely to result from mutations in the human orthologs [24].

Our overarching goal is to better understand phenotypic effects of nsSNVs on the active site of enzymes. Although we use a small set of enzymes here to perform a preliminary proof of principle, this experiment lays the foundation for a method of cellular modeling that will move toward an “omics” approach with potential predictive ability. Similar large-scale studies already demonstrate the utility of this type of approach to predict metabolite concentrations related to environmental conditions [25], enzyme phosphorylation [26] and even whole gene deletion [27], but to the best of our knowledge there is no such effort to apply a metabolomics approach to the analysis of active-site- nsSNV-containing proteins to yield predictive and/or diagnostic information.

## Results and discussion

The complete human and yeast proteomes, and all protein subsets, available from UniProtKB/Swiss-Prot, provide curated annotation information regarding both nsSNV and active sites [28]. The dbSNP database [29] provides additional information on variations and the Conserved Domain Database (CDD) [30] provides additional information on active sites. The SNVDis tool uses this information to return an output table with all human proteins containing nonsynonymous substitutions at the active site.

Note here that we do not discriminate between heterozygous and homozygous nsSNVs because pertinent information is unavailable for the majority of human variation data. Previous findings imply that most nsSNVs in active sites are rare or heterozygous because they are expected to be selected against and, therefore, unlikely to be observed in a homozygous pair [31]. Studies of consanguineous families may prove a good source for detection of homozygous active site nsSNVs in the future, but there are currently few documented cases [32] of catalytic homozygote variants. This is problematic as currently available models assume the haploid condition of a yeast cell. Results of reducing the flux to zero in such a model cannot be easily generalized to a diploid, heterozygous state unless known loss of function via haploinsufficiency or dominant negative phenotypes is established. Although we cannot say with certainty that the variations presented in this paper confer this phenotype, there is literature evidencing the heterozygous loss of function associated with active site mutations in a number of mammalian genes including mouse DNA polymerase δ [33], human DNA polymerase γ [34] and mammalian 11β-hydroxysteroid dehydrogenase type I [35]. Furthermore, the aforementioned murine study was extended from studies of homologous mutations in haploid yeast. Thus, while we cannot fully extend haploid modeling to proteins in diploid organisms, we suggest that yeast enzymes whose knockouts alter flux balance necessitate further attention be given to their orthologous human counterparts, for both homozygous and heterozygous variations. Specifically, we propose that haploid models could still provide quantitative value toward the development of predictive assays for highly conserved pathways in cases of true loss of function due to heterozygous mutation at human active sites.

A proteome-wide search in SNVDis returns 559 unique human proteins (Additional file 1: Table A1) with nsSNV at the active site. Subsequent pathway analysis using annotations from KEGG shows a subset of 34 proteins (updated from previous publication to include two additional proteins from the same set of 559 which were later found to meet the criteria) with nsSNV at the active site and a 1 enzyme:1 pathway:1 reaction relationship. Full methods and discussion for these and other preliminary results can be seen in the prior publication.

### Reciprocal best BLAST hits method to identify human-yeast ortholog pairs

To identify proteins from yeast (*S. cerevisiae)* orthologous to the 559 active-site-nsSNV-containing human proteins, the reciprocal best BLAST hits method was used. The sequences for the 559 active-site-nsSNV-containing human proteins were extracted from UniProtKB/Swiss-Prot by UniProt Accession and used as the query in a BLAST [36] against the yeast proteome. Of the 559, 173 proteins have no hits against the yeast proteome with E-value less than 0.0001 and 146 proteins have significant hits but are deemed to be paralogous. Thus, 240 of the 559 human proteins are considered to have a one-way best hit. These 240 best-hit yeast proteins were then used as the query in a second BLAST against the entire human proteome. Of these 240, 127 have a unidirectional best-match such that the highest scoring yeast match from the human BLAST against the yeast proteome has an alternative highest-scoring human match when queried against the entire human proteome. This leaves 113 proteins with active site nsSNV to have a reciprocal best-match ortholog in yeast (Table 1, full reciprocal best BLAST hits data can be shown in Additional file 1: Table A2).

**Table 1:** Human proteins with active site nsSNV and their yeast ortholog match

**Human protein**	**Yeast ortholog**	**Human protein**	**Yeast ortholog**	**Human protein**	**Yeast ortholog**
Q9GZR2	Q08237	Q9P2J9	Q12511	Q15067	P13711
P49247	Q12189	Q9BZP6	Q06350	Q96S44	P53323
Q9UBZ4	P38207	O60825	P32604	O95154	P42884
Q14410	P32190	O15305	P07283	P78368	P23292
P51659	Q02207	Q9UNI6	Q02256	Q6PI48	P15179
P20618	P23724	Q96RR4	P43637	Q13164	Q00772
Q96T52	P46972	Q9NUW8	P38319	P06744	P12709
P08397	P28789	P00480	P05150	P11498	P32327
P04424	P04076	Q6IA69	P38795	P05186	P11491
P40818	P32571	Q04760	P50107	Q8TF76	P32789
Q8IWW8	P10127	P36873	P32598	P29120	P13134
P37268	P29704	Q9P2K8	P15442	Q8IWX5	P23501
Q96G46	Q06053	Q96GX9	P47095	Q96DP5	P32785
P48637	Q08220	Q13332	P25044	Q9Y3E5	P34222
P09467	P09201	P12955	P43590	P25789	P23638
Q9UGM6	P04803	P06132	P32347	P60484	P53916
P78549	P31378	O75191	P42826	P49917	Q08387
O96017	P39009	Q9H3S4	P35202	P32320	Q06549
Q7L3T8	P39965	P06737	P06738	Q9NSY1	P53974
Q9NYY3	P32562	Q13907	P15496	O60942	Q01159
O43426	P50942	P49841	P38615	Q9UGP5	P25615
O95363	P08425	P36871	P37012	Q9BUP3	P40008
P34949	P29952	Q9UBZ9	P12689	P54098	P15801
Q7L211	P42840	Q9HBY8	P12688	Q9UJM8	P00175
P07741	P49435	P07902	P08431	Q8WUX2	P32656
P13716	P05373	Q9Y2L1	Q08162	Q14249	P08466
P48449	P38604	Q96C11	Q04585	Q5VTY9	Q08929
P49336	P39073	O14818	P40303	Q86YJ6	P16120
Q8IZ73	Q12362	O00743	P20604	P12081	P07263
Q69YN2	P53255	Q5T2R2	P18900	Q9UQB9	P38991
Q99447	P33412	P04180	P40345	O14734	P41903
P30793	P51601	Q9Y3Q0	P47161	P14550	P14065
Q9BV23	P53750	O43175	P40054	P08243	P49090
P56937	Q12452	P22830	P16622	Q86V88	P40081
Q16769	P43599	O95336	P38858	Q8IXB1	P40564
Q15386	P53119	Q969P6	P04786	Q9Y3B8	P54964
Q9Y2H1	P53894	Q9Y2Z4	P48527	P00813	P53909
Q9UI42	P38836	Q9Y6R4	P53599		

This table lists the pool of 113 reciprocal best match human-yeast ortholog pairs, a subset from the original 559 proteins with active site nsSNV.

### Identification of candidate enzymes for in vivo validation

To be considered ideal for laboratory validation, we want to first limit our consideration to the proteins in Table 2 which have a simple 1 enzyme:1 pathway:1 reaction relationship relationship. This 1:1:1 ratio drastically limits the possibility for an enzyme to affect or be affected by multiple metabolites during laboratory experiments. Of the 34 potential candidates, 11 had no best hit and 10 had a one-way best hit, leaving 11 proteins as potential candidates with reciprocal best-match orthologs.

**Table 2:** 34 proteins with active site nsSNV with substrate/product/pathway relationships ideal for use in metabolomics

**UniProtKB**	**Normal**	**Variation**	**Position**	**Substrate increased**	**Substrate ID**	**Product decreased**	**Product ID**
**A2RTX5**	t	a	532	L-threonine	CID: 6288	L-threonyl-tRNA	SID: 5901
**O43175**	r	c	236	3-phospho-D-glycerate	CID: 439183	3-phosphonooxypyruvate	CID: 105
**O75891**	p	l	107	10-formyltetrahydrofolate	CID: 122347	Tetrahydrofolate	CID: 91443
**O75908**	h	q	434	cholesterol	CID: 5997	Cholesterol ester	SID: 5537
**O75936**	h	q	347	4-trimethylammoniobutanoate	CID: 134	Carnitine	CID: 85
**O95336**	r	c	185	6-phospho-D-glucono-1,5-lactone	CID: 439452	6-phospho-D-gluconate	CID: 91493
**O95363**	q	x	160	L-phenylalanine	CID: 6140	L-phenylalanyl-tRNA(Phe)	SID: 6321
**O95479**	r	c	728	Beta-D-glucose 6-phosphate	CID: 439427	6-phospho-D-gluconate	CID: 91493
**O95861**	a	v	221	PAPS	CID: 10214	Adenylyl sulfate	CID: 10238
**P04180**	s	n	205	Phosphatidyl choline	SID: 3457	2-lysolecithin	SID: 6900
**P09417**	y	c	150	Tetrahydrobiopterin	CID: 44257	Dihydrobiopterin	CID: 133246
**P13716**	r	p	221	5-aminolevulinate	CID: 137	Porphobilinogen	CID: 1021
**P22830**	d	g	383	Ferrous ion | protoporphyrin IX	CID: 27284 | CID: 4971	Heme	CID: 444097
**P23109**	h	q	305	Adenylic acid	CID: 6083	Ammonia | inosinic acid	CID: 222 | CID: 8582
**P23946**	h	r	66	Angiotensin I	CID: 3081372	Angiotensin	CID: 172198
**P25092**	t	a	618	Guanosine 5'-triphosphate	CID: 6830	Cyclic gmp	CID: 24316
**P42357**	g	s	165	L-histidine	CID: 6274	Urocanic acid	CID: 736715
**P43251**	a	s	271	Biocytin	CID: 440721	Biotin | L-lysine	CID: 171548 |CID: 5962
**P48637**	r	c	125	Glycine | gamma-glutamylcysteine	CID: 750 | CID: 123938	Glutathione	CID: 124886
**P49247**	d	y	160	D-ribulose 5-phosphate	CID: 439184	Ribose 5-phosphate	CID: 439167
**Q02127**	t	i	284	L-dihydroorotic acid	CID: 439216	Orotic acid	CID: 967
**Q02809**	r	h	718	Protein lysine	SID: 5259	Procollagen 5-hydroxy-L-lysine	SID: 4433
**Q16873**	r	k	104	Leukotriene A4	CID: 5280383	Leukotriene C4	CID: 5280493
**Q3SY69**	y	x	127	10-formyltetrahydrofolate	CID: 122347	Tetrahydrofolate	CID: 91443
**Q6PI48**	r	x	266	L-aspartic acid	CID: 5960	L-aspartyl-tRNA(Asp)	SID: 5893
**Q8IVS2**	s	t	153	Malonyl-CoA	CID: 10663	Malonyl-[acyl-carrier-protein]	SID: 4431
**Q8N5D6**	y	x	121	Globoside	SID: 124490726	IV3GalNAca-Gb4Cer	SID: 124490727
**Q8TDQ7**	p	s	182	D-glucosamine phosphate	CID: 440997	Beta-D-fructose 6-phosphate	CID: 440641
**Q96C23**	r	x	82	Alpha-D-glucose	CID: 79025	Beta-D-glucose	CID: 64689
**Q96GX9**	g	r	47	S-methyl-5-thio-D-ribulose 1-phosphate	CID: 174549	5-(methylthio)-2,3-dioxopenyl phosphate	CID: 561
**Q99487**	h	r	314	Platelet-activating factor	SID: 7195	1-alkyl-sn-glycero-3-phosphocholine	SID: 6975
**Q9NVF9**	n	s	94	Ethanolamine	CID: 700	Phosphoethanolamine	CID: 1015
**Q9P2T1**	g	d	242	Guanylic acid	CID: 6804	Inosinic acid	CID: 8582
**Q9Y2Z4**	g	s	243	L-tyrosine	CID: 6057	L-tyrosyl-tRNA(Tyr)	SID: 5781

This table provides the UniProtKB accession ID, normal and variable residues, position of the variation and substrate/product pairs with PubChem IDs for 34 proteins with annotated nsSNVs at the active site. These proteins were chosen for this study based on a one-protein, one-variation, one-substrate/product pair relationship to simplify preliminary modeling by ensuring no complex interactions for the given protein pathway. NOTE: This table has been updated since the prior publication to include 2 additional proteins which were later identified to meet the criteria.

We must also consider the case when the product of an enzymatic reaction can be synthesized by alternative means. For this paper, we decided to consider these cases due to literature supporting biochemical importance of dysfunction despite multiple pathways to product.

**Figure 1:**
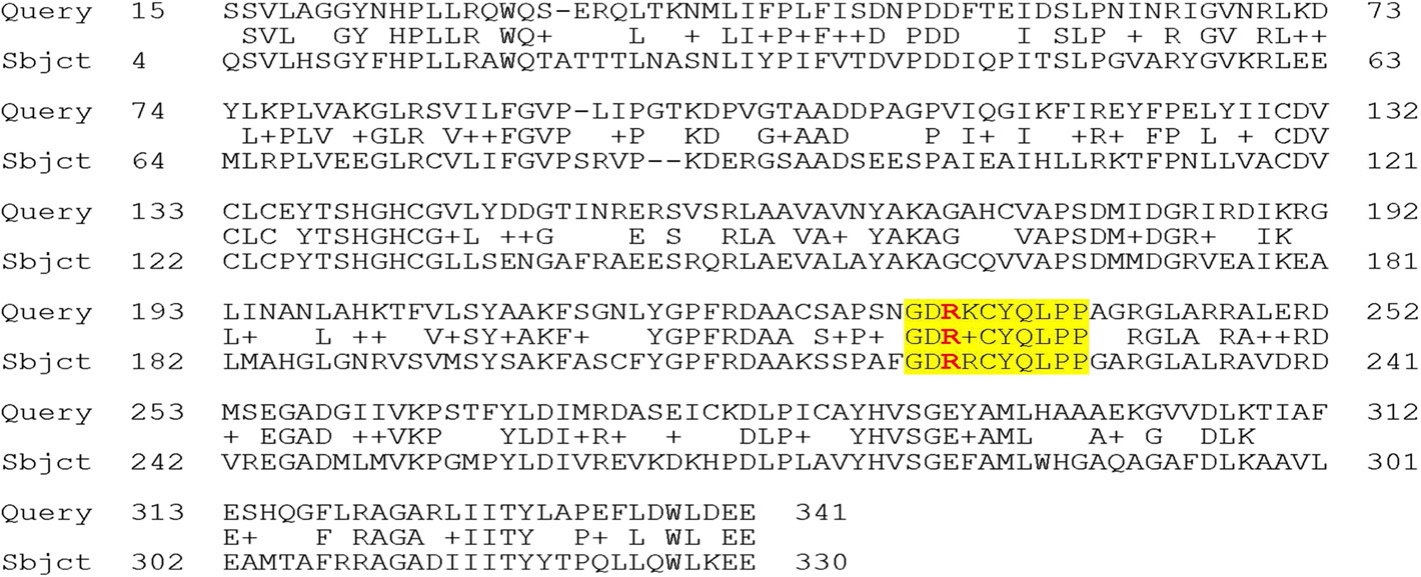
**Manual verification of residue conservation at the active site in both human and yeast orthologs.** This is the reciprocal best BLAST hits alignment using yeast protein P05373, Delta-aminolevulinic acid dehydratase for *S. cerevisiae*, as the query searching against the entire human proteome. The only human hit is P13716, also delta-aminolevulinic acid for *H. sapiens*. From genbank annotations, we know the active site should occur at position 221 in the human sequence, corresponding to position 232 in the yeast sequence. Here we see conservation not only among the active site residues, but also in the surrounding region, which can also be important in facilitating active site binding.

Additionally, we want to ensure the variable site of interest, the active site, is conserved across ortholog pairs. If functional residues are not conserved, we cannot say that experimental observations resulting from a variation in a yeast enzyme is at all indicative of outcomes of variation in the corresponding human enzyme. This was checked by manual examination of alignments (Figure 1). The minimum requirement for active site conservation is that the residue(s) annotated as the active site residue(s) are identical across species, although many of the human-yeast pairs have blocks of up 10 amino acids conserved across species. 10 of the 11 ortholog pairs were conserved at the active site.

We want to further limit wet-lab candidate enzymes to those with no other orthologs or paralogs. Even though the proteins under consideration at this point are reciprocal best-matches, proteins with closely related homologs limit the confidence with which we can report results of laboratory validation experiments. For example, if a human protein has a best match with a yeast protein which has paralogs, we cannot be sure upon knockout of the enzyme that the paralogous proteins will not interfere in the metabolism we are trying to assay. Exclusion of proteins with orthologs and paralogs was performed by manually examining alignments. This exclusion now reduces our list of potential candidates to 6. Table 3 lists the active site residue and surrounding conserved residues for these 6 proteins.

**Table 3:** Conserved residues between human and yeast orthologs at and around the active site

**UniProtKB**	**Yeast AC**	**Human active site**	**Yeast active site**	**Conservation block**
P13716	P05373	221 R	232 R	GDR + CYQLPP
P22830	P16622	383 D	361 D	+ADLV
P48637	Q08220	125 R	128 R	RSDY+
P49247	Q12189	160 D	107 D	DGADEVD
Q96GX9	P47095	47 G	39 G	TGTGGGIS + K
Q9Y2Z4	P48527	243 G	261 G	+Q + GG + DQ

**Figure 2:**
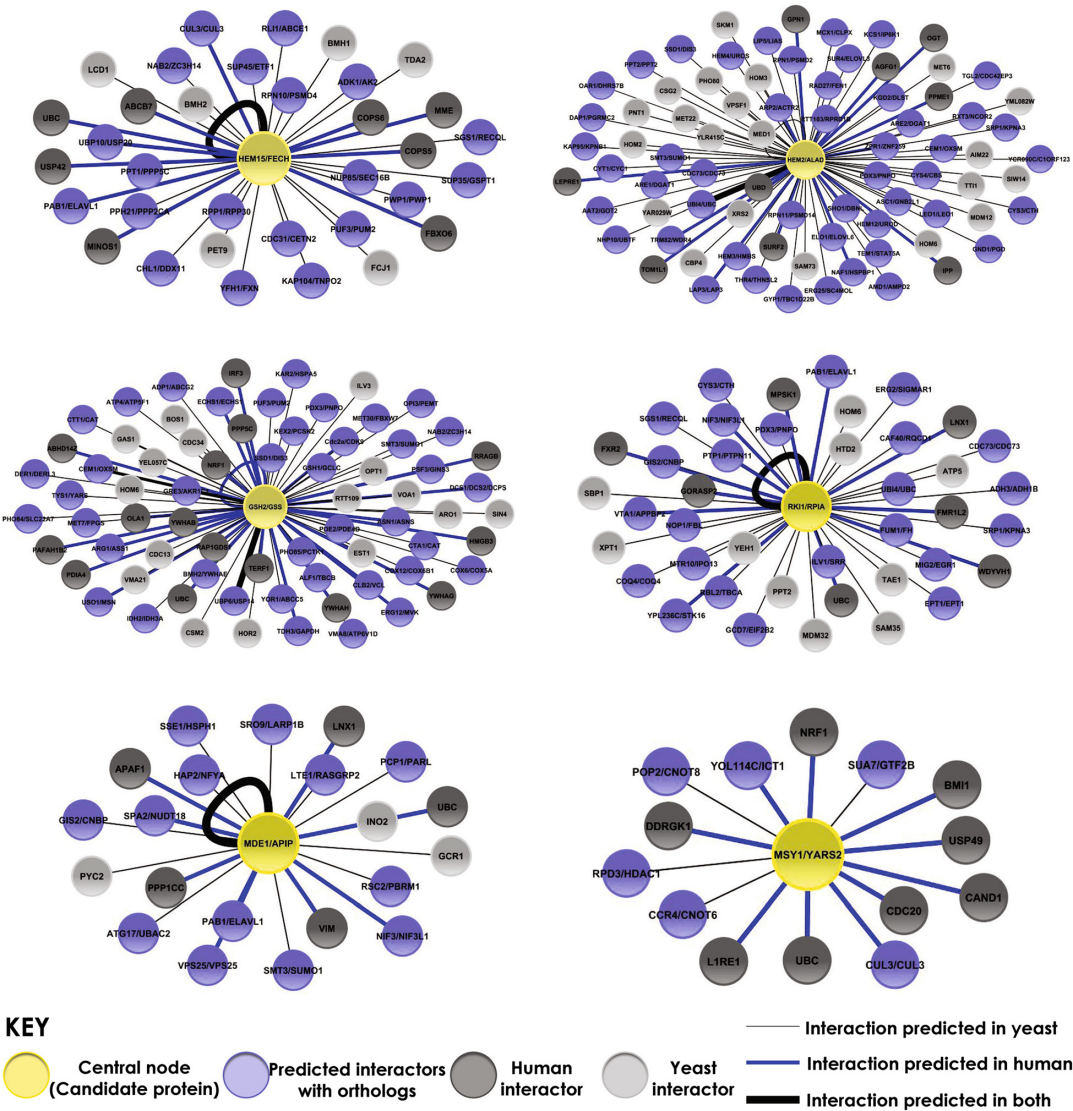
**Similar protein interactions between human and yeast counterparts.** Known and predicted protein interactions were retrieved from IntAct [39] and BioGrid [40] using both human and yeast gene names as query terms. Interactions were organized and edited following suggestions by Lim et al. [37]. It is interesting to note that although a sizeable portion of predicted interactors are also predicted to have functional orthology between species, very few of the inter-protein interactions among predicted orthologs were reported for both species.

**Figure 3:**
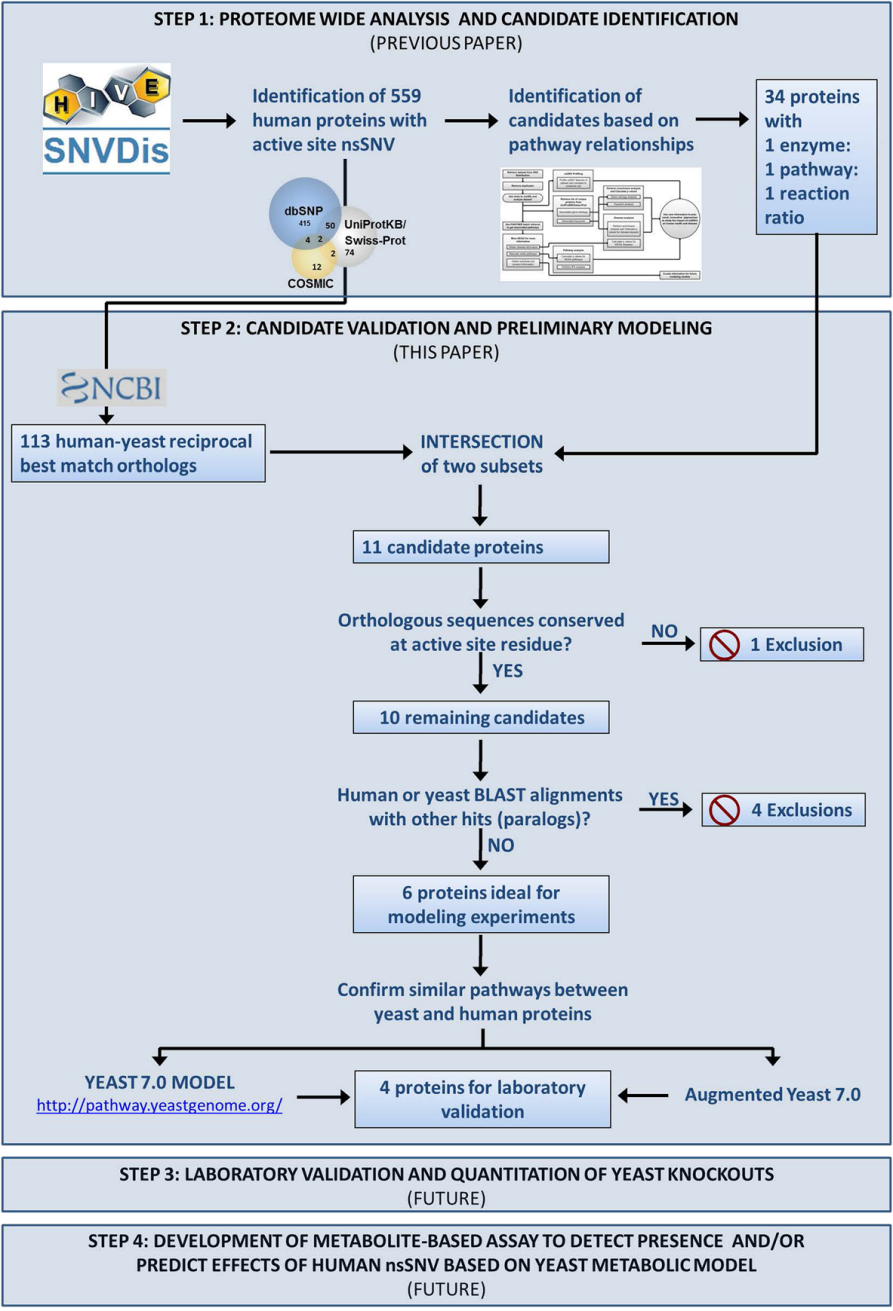
**Schema of identification of candidate proteins for yeast modeling.** The overall method used in this paper was manually performed, but consists of simple steps and queries which could easily be automated for the performance of future similar studies.

Finally, we want to make sure the ortholog pairs are involved in similar pathways. If a human protein has diverged to act on different metabolites we again cannot use its yeast ortholog as a model to understand effects of variation in human proteins. Pathway information for the human proteins was previously retrieved and summarized in Table 2. Pathway information for the yeast proteins was similarly retrieved from KEGG and recorded. Pathway conservation is indicated by similar pathway map with identical substrates and products. All 6 ortholog pairs were found to participate in the same pathways, acting on the same substrates and yielding the same products. Pathway information for these 6 proteins is included in Table 4. Further support for functional similarity can be seen in Figure 2, showing evidenced interactions (collected and curated following guidance by Lim et. al. [37] with other proteins and the distribution of functional human-yeast orthologs predicted by Isobase [38] among these subsets. A summary schema of the overall method used in identification of these 6 candidate proteins is presented in Figure 3.

**Table 4:** Pathway comparison between human candidate proteins and yeast orthologs using KEGG pathway annotations

**Human UniProt Ac**	**Gene name**	**Pathways**	**Yeast UniProt Ac**	**Yeast gene name**	**Pathway match?**	**Details**
P13716	ALAD	Porphyrin and clorophyll metabolism	P05373	HEM2	Yes	Same
		Biosynthesis of secondary metabolites			No	Pathway only annotated in yeast; same substrate/product
P22830	FECH	Porphyrin and clorophyll metabolism	P16622	HEM15	Yes	Same
		Biosynthesis of secondary metabolites			No	Pathway only annotated in yeast; same substrate/product
P48637	GSS	Glutathione metabolism	Q08220	GSH2	Yes	Same
P49247	RPIA	Pentose phosphate pathway	Q12189	RKI1	Yes	Same
		Biosynthesis of secondary metabolites			No	Pathway only annotated in yeast; same substrate/product
Q96GX9	APIP	Cysteine and methionine metabolism	P47095	MDE1	Yes	Same
Q9Y2Z4	YARS2	Aminoacyl-tRNA biosynthesis	P48527	MSY1	Yes	Same

This table summarizes the pathway involvement of the six identified candidate proteins. For all 6 proteins, the primary pathway involvement is the same, down to the same products and substrates and the same place within a pathway. For three of the yeast proteins, however, the pathway annotation in KEGG listed an additional pathway not annotated for humans: “Biosynthesis of secondary metabolites.” This is likely due to differential annotation between yeast and humans within KEGG. Because the substrates and products annotated for these pathways were identical to those of the primary pathways, these three proteins were not excluded and went onto analysis in the yeast FBA modeling.

### Yeast 7.0 COBRA modeling

Five of the six identified candidate enzymes were input into the Yeast 7.0 model available at http://pathway.yeastgenome.org/[17]. Yeast methylthioribulose-1-phosphate dehydratase (MDE1), P47095, had no corresponding gene in the model and was therefore excluded from this analysis. When knockouts are generated in these genes, they all show normal growth rates except ribose-5-phosphate isomerase (RPIA),Q12189, which showed no growth. However, Yeast 7.0 is not equipped to predict growth for all of these reactions because some important compounds and cofactors are not currently accounted for in the model. Findings are summarized in Table 5. (Full output available in Additional file 1: Table A3).

**Table 5:** Summary of FBA results

**Human**	**Yeast**	**Reaction tested**	**Yeast 7.0 results**	
			**Knock-out growth**	**Explanation**
P13716	P05373	Porphobilinogen synthesis	No FBA growth	Ferroheme b + Yeast 7.0 biomass equation
P22830	P16622	Protoheme ferrochelatase	No FBA growth	Ferroheme b + Yeast 7.0 biomass equation
P48637	Q08220	Glutathione synthesis	WT growth (μ = 0.1405 hr^−1^)	0 flux through reaction in WT (r_0485)
P49247	Q12189	Ribose-5-phosphate isomerization	No FBA growth	Phosphoribosyl-pyrophosphate block
Q96GX9	P47095	Methylthioribulose 1-phosphate dehydratase	Not determined	Gene not present in Yeast 7.0 model
Q9Y2Z4	P48527	Mitochondrial tyrosine-tRNA-ligase reaction	No FBA growth	Mitochondrial tRNA requirement + Yeast 7.0 biomass equation

P47095 impact could not be modeled as the Yeast 7.0 model contains no corresponding gene.

### Modified yeast 7.0 modeling with updated biomass function

Under the consideration that a modeling tool with a more comprehensive biomass function could provide more information, we repeated modeling of the 5 relevant enzymes using a biomass function with added requirements for ferroheme b and charged tRNA(Tyr) in the mitochondrion. This model was able to implicate three additional proteins with function in essential reactions: delta-aminolevulinic acid dehydratase (ALADH), P05373; mitochondrial ferrochelatase, P16622; and mitochondrial tyrosine-tRNA ligase, P48527. This additional examination, in conjunction with sequence and pathway conservation, supports our argument for inclusion of the pertinent pathways in yeast cell models. A summary of the findings can be seen in Table 5. (see Additional file 1: Table A3 for full output).

The above Yeast 7.0 results, combined with literature support, sequence and pathway annotations, maintain our hypothesis that we should be able to use yeast orthologs with active site conservation to model the effect of a variation at the active site of its human ortholog in order to obtain a predictive overview of altered enzyme activity resulting from the variation. Furthermore, we see the potential for the utility of this method to improve as advanced modeling efforts continue to progress.

### Biomedical relevance

We hypothesize that nonsynonymous variation in an enzyme’s active site should have deleterious effects on the enzyme’s activity and function. Although only one of the six human enzyme SNPs has a direct link to the Online Mendelian Inheritance in Man® database (OMIM®) [41] in dbSNP (rs28936396 in P48637, human glutathione synthetase linked to OMIM 266130 – Glutathione synthetase deficiency), all six have literature related to dysfunction and disease caused by variants found at other positions within the protein sequence. If our assumptions regarding active site variation are correct, then nsSNVs at the active site of enzymes involved in metabolic syndromes will likely alter protein function and contribute to similar syndromes. Furthermore, yeast orthologs for these enzymes with strong pathway and active site conservation may be used to verify the metabolic effects of such variation with respect to metabolite concentrations. Although the current Yeast 7.0 model supports this hypothesis for RPIA alone, we argue for inclusion of the pathways and metabolites associated with these proteins in future yeast models based on consideration of the augmented model findings, literature and conservation among orthologs. Thus, we present five potential disease-related proteins with active site nsSNVs (along with yeast orthologs) in need of laboratory experimentation to validate the veracity of disease related to the SNV.

### Human P49247/Yeast Q12189

Ribose-5 phosphate isomerase (RPI) participates in the pentose phosphate pathway (PPP), an alternative pathway for glucose oxidation accounting for up to 20% of glucose oxidation in normal tissue. [42] Synthesis of ribose 5-phosphate from ribulose 5-phosphate via RPI is required for nucleotide and nucleic acid synthesis, and participates in the downstream production of glycolytic intermediates [43]. With reduced RPI activity, ribose 5-phosphate becomes unavailable and the necessary glycolytic metabolites are not produced. Enzyme deficiency results in clinical symptoms of leukoencephalopathy and peripheral polyneuropathy. The RPIA gene has also been identified to be hypermethylated at CpG sites in breast cancer [44].

As of 2004, RPI was only the second known inborn error in the reversible portion of the PPP [45]; the PPP connects pentose phosphates to glycolytic intermediates. Furthermore, there are currently only 2 mutations listed in the Human Gene Mutation Database [46] (accessed 8/22/13) for the RPIA gene, neither of which is the aspartic acid to tyrosine variation at active site position 160 reported here (rs11549730). RPI has been observed to be variably conserved among species ranging from 52% similarity between human and *E. coli* to strong conservation noted between human and other mammals [47] and RKI1 disruption has been shown to be deleterious to yeast growth [48].

### Human P13716/Yeast P05373

Delta-aminolevulinic acid dehydratase (ALADH), encoded by the ALAD gene in humans, catalyzes the first common step in heme and other tetrapyrrole biosynthesis [49] and has also been observed to interact with the proteasome [50]. The wild-type enzyme exists as a high-activity homooctamer, but alternative lower-activity hexamer assemblies have been found [51]. In fact, naturally occurring ALAD porphyria-associated human variants have been shown to have an increased susceptibility to hexamer-stabilizing inhibitors. Lead-poisoning is also hallmarked by reduced ALAD activity. 5-aminolevulinate accumulates as a result of the lowered activity and is responsible for the toxic effects observed in both disease states. It has been suggested that small molecule stabilization of alternate oligomers (morpheeins) can be used as a therapy to target delta-aminolevulinic acid dehydratase [52], in addition to human immunodeficiency virus integrase, tumor necrosis factor α, mammalian ribonucleotide reductase and other disease-related proteins for which there is evidence of alternate, functionally differential oligomers [53].

Mutants in the corresponding yeast HEM2 gene have been found to disrupt heme biosynthesis, requiring provision of heme for growth in culture [54]. Yeast and human ALADH differ with respect to Zn cofactor binding, thus care is required to ensure homology models account for this difference in stoichiometry [55]. However, comparison of primary sequences across a diverse array of species shows a strong degree of similarity in the region of the active site and a zinc-binding motif [56].

There are many studies discussing ALAD-porphyria associated mutations [57,58] but responsibility for the disease state has not yet been assigned to the deletion at nucleotide position 843. Interestingly, this deletion in the active site codon also results in a frameshift, further complicating comprehension of potential disease mechanism(s).

### Human P22830/Yeast P16622

Encoded by the FECH gene, ferrochelatase is the final enzyme in heme biosynthesis, catalyzing the formation of protoheme IX from protoporphyrin IX and ferrous iron [59]. Ferrocheletase is important to human health due to the critical and diverse functions of its product cofactor, heme. Defects in this enzyme have been related to erythropoietic protoporphyria (EPP). EPP is a relatively benign disease with predominant manifestation of photosensitivity, although it has been reported with liver complications in 2-5% of patients [60]. Biological findings include elevated concentrations of protoporphyrin in erythrocytes, plasma and feces, and a large amount of protoporphyrin in the skin [61].

Comparisons between ferrochelatases of multiple species reveal the proteins belonging to human, mouse, chicken, frog and *Drosophila melanogaster* to be metalloenzymes with a [2Fe-2S] cofactor, thought to play a structural stabilizing role in humans [62]. The corresponding bacteria, yeast and plant proteins contain no iron-sulfur center, but the ferrochelatase from *S. cerevisiae* is similar in other respects to eukaryotic ferrochelatases. Yeast mutants with total or partial FECH activity deficiency and resultant protoporphyrin accumulation have been isolated [63].

### Human Q9Y2Z4/Yeast P48527

Aminoacyl-tRNA synthetases (ARSs) catalyze the linkage of specific amino acids to their cognate tRNAs [64]. Mitochondrial tyrosyl-tRNA synthetase (TyrRS) is responsible for charging tyrosine to its tRNA. Although belonging to class I synthetases, tRNA recognition occurs in a way more common to class II ARSs [65]. Defects in TyrRS are associated with myopathy with lactic acidosis and sideroblastic anemia 2 (MLASA2). It has been proposed that diminished aminoacylation activity of the enzyme leads to decreased mitochondrial protein synthesis and mitochondrial respiratory chain dysfunction [66]. TyrRS also has known involvement with cell-signaling [67] and angiogenesis [68]. Although there are multiple known disease-related TyrRS mutants, it is unclear for some mutants whether pathogenicity results from dysfunction in aminoacylation or cytokine activation.

Alignments with TyrRSs from several species reveals 34% identity with yeast and similarly low identity across the board, with greatest conservation confined to the N-terminus [69]. Gly-244 and Asp-246 are conserved among class I synthetase catalytically important residues, but a total of 8 residues are known to participate in hydrogen bonding [70].

### Human Q96GX9/Yeast P47095

Methylthioribulose-1-phosphate dehydratase, encoded by the APIP (APAF1-interacting protein) gene, catalyzes the dehydratase step of the methionine salvage pathway, converting methylthioribulose-1-phosphate into 2,3 dioxomethylthiopentane-1-phosphate [71]. The methionine salvage pathway recycles sulfur metabolites by recovery of methionine from methylthioadenosine (MTA). This pathway plays a critical role in many biological functions including apoptosis, inflammation and cancer [72].

APIP is highly expressed in skeletal muscle and known for an anti-apoptotic role [73]. One common APIP SNP was observed to reduce enzyme activity, resulting in an increase in cell death in response to *Salmonella*[74].

Currently excluded from the Yeast 7.0 model, the MDH1 enzyme is of critical importance to the pathway. Loss of function leaves the cell with no isozymes for methionine salvage, as demonstrated in the YeastCyc pathway/genome database (PGDB) within BioCyc [75]. The enzyme and the entire methionine salvage pathway seem to be functionally conserved in yeast [76] and should therefore serve as an adequate model in yeast experimentation to validate predicted loss of function due to the contained nsSNV.

## Conclusions

The method proposed by this series of studies can be generalized to identify all proteins affected by variation at functionally important residues, to determine the availability of orthologous yeast proteins to model the effects of such variation and, in the future, to predict and quantify cellular and potentially organismal responses to the variation. The specific analyses presented here for demonstration of the method further demonstrate that nonsynonymous single nucleotide variation at the active site should disrupt enzyme function and potentially induce disease in yeast. However, to fully and confidently extrapolate this finding to humans, disruption of enzyme function must be checked against the effects of zygosity. This is currently a limitation imposed by existing data due to the lack of relevant frequency information for the majority of the variations studied. We have provided 6 sets of human/yeast enzyme ortholog pairs for which the human protein has been experimentally shown with nsSNV at the active site and the yeast enzyme has been verified to share important conserved properties. Each of these enzymes is known to have deleterious effects on human physiology when dysfunctional, but the loss of function has been directly related in OMIM to only one of the active site variants, glutathione synthetase. Thus, we present five viable candidates for variant-related disease discovery. Preliminary FBA modeling shows predictive support for one of these enzymes, ribose-5-phosphate isomerase. Experimental validation of these and similar findings will continue to increase the knowledge universe, in turn facilitating the development of more powerful, robust models. Advances in the technology employed by this method may enable the long-term creation of metabolite profiles associated with active site variation for corresponding human and yeast orthologs. Simple biochemical assays comparing human and yeast metabolite concentrations to the profiles could facilitate detection of active site non-synonymous variation. This may further lay the foundation for future diagnostic pipelines to predict unknown outcomes of active site nsSNVs in these and other enzymes.

## Methods

### Datasets and annotated information

Proteomes and all protein subsets were retrieved from UniProtKB/Swiss-Prot (UniProt release 2012_06 [28]. nsSNV data were extracted from UniProtKB/Swiss-Prot, dbSNP (build 135) [29], COSMIC (version 59) [77] and NCI 60 [78] (accessed June 28, 2012). The SNVDis tool (https://hive.biochemistry.gwu.edu/snpdis.cgi?cmd=dmSnpdis) [12] was used to retrieve the set of all proteins with nsSNVs at the active site (as annotated by UniProtKB/Swiss-Prot or CDD). Pathway information was retrieved from Kyoto Encyclopedia of Genes and Genomes (KEGG) [79] using the Protein Analysis Through Evolutionary Relationships (PANTHER) Classification System [80]. Substrate, product and disease information was gathered manually from KEGG and UniProtKB/Swiss-Prot. Official names and PubChem [81] Compound IDs (CIDs) for compounds with specific chemical structures, or Substance IDs (SIDs) for those without, were recorded for all substrates and products.

### Analysis

#### Reciprocal best BLAST hits method

Stand-alone BLAST (version 2.2.27+) [82]was downloaded from the NCBI website. The yeast (*S. cerevisiae*) proteome was downloaded from Uniprot by using the term “Baker's yeast [559292]” for the field of Organism[OS], combined with the term “Complete proteome”’ in the Keyword field. The set of 559 previously selected human proteins was retrieved from Uniprot/Swis-protKB in batch mode using the corresponding accession numbers.

The yeast proteome file was indexed as the database, and the human protein dataset was used as the query for the nucleotide-to-nucleotide Blastn algorithm with all default parameters and E-value higher than 10^−5^. The customized script was developed to extract best hits from the Blastn result. All the yeast proteins that met the criteria were batch-retrieved from UniprotKB/Swiss-prot by accession numbers and prepared for the opposite direction BLAST. Reciprocal best BLAST hits method was then accomplished by using the selected yeast proteins as the query set to align against the indexed human proteome database downloaded from Uniprot following the similar method described above. A human-yeast ortholog table was generated via a customized script parsing the two-way BLAST results. Thus, only those proteins which are mutual best hits with p-value higher than 10^−5^ were marked as the ortholog.

#### Manual identification of orthologous pairs

Manual examination of BLAST alignment results was performed to verify residue conservation at and around the active site, as well as exclusion of proteins with additional homologs. Residue conservation was observed for all 6 candidate enzymes and reported as the longest continuous sequence conservation block containing the active site residue. Confirmation of active site annotation across species was achieved by consulting relevant databases. Manual confirmation of single-best-match orthologs was done by visual verification of BLAST results of corresponding human and yeast proteins performed separately with human and yeast as query species.

#### FBA procedures

COBRA simulations of the Yeast 7.0 genome-scale model were performed using Yeast 7.0.0 in the COBRA Toolbox 5.0.0 within MATLAB R2010a. In order to simulate the effects of knocking out genes YGL040C, YOR176W, and YPL097W, we added small (0.0005 mmol/gCDW/hr) requirements for ferroheme b and charged tRNA(Tyr) in the mitochondrion to the Yeast 7.0 biomass function, creating an augmented biomass function. Experimental evidence for these deletions drawn from the Saccharomyces Genome Database [83] suggests that YGL040C and YOR176W null mutants are inviable in large scale surveys, and that YPL097W mutants are deficient in respiration. Reference growth rates for wild-type cells lacking gene knockouts were obtained based on aerobic growth in glucose minimal media, using both the augmented and the original Yeast 7.0 biomass function. Additional file 1: Table A3 contains fluxes obtained for simulations of wild-type cells using the augmented biomass function and are labeled as ‘augmented_flux’, while fluxes obtained for simulations of wild-type cells using the Yeast 7.0 biomass function are labeled as ‘7.00_flux’. Gene knockouts were simulated by limiting flux through the reaction carried out by the gene product in question to zero, equaling a total loss of function for that reaction. All COBRA simulations employed taxicab norm minimization of fluxes as described in the documentation for the optimizeCbModel function.

## Reviewers' comments

We appreciate the reviewer’s comments from Dr. Daniel Haft and from Dr. Igor B. Rogozin. We have revised the manuscript according to your comments and suggestions.

### Reviewer 1: Dr. Daniel Haft

Previous work by this group identified 559 human enzymes in which missense mutations replace an enzyme’s active site residue in rare alleles. Of these, 34 are assigned a single enzymatic activity rather than a panel of alternate substrates, with the reaction belonging to a single pathway in human as documented by KEGG. For this paper, the collection is winnowed further, to count only those for which bi-directional best BLAST matches between human and yeast identify putative ortholog pairs, uncomplicated by paralogs that could overlap in function. This stringent filter gets the list down to six enzymes, the topic of this paper. A tool for flux balance analysis (FBA) is available for yeast, but not for human. Thus, for these six enzymes, computation based on a yeast metabolic model leads to predictions that loss of the same function in human causes disease.

In one sense, there is no surprise here. Mutations away from the active site already were known to cause human disease for five of the six enzymes. Loss of the active site residue should be no less catastrophic to function than truncation or frameshift errors. It is possible that a heterozygous active site mutant could produce a currently unrecognized form of a disease now known only for homozygotes of an incompletely inactivated enzyme, but that is not the point of the paper. The yeast model is haploid. Presumably, the implications are for homozygotes of the mutant form in human.

Authors’ response: *Perhaps it wasn’t clear from our original discussion, but we agree that the haploid yeast model is only suited to truly “model” homozygous cases. However, we can envision potential scenarios in which a haploid model could still provide predictive quantitative value in assays in highly conserved pathways for loss of function due to heterozygous mutation at active sites in humans. We did revisit the text relevant to this issue and modified it to improve clarity.*

Furthermore, we are not claiming that homozygous alleles are altogether too rare, in fact the journal article we cite here (Ng PC1, Levy S, Huang J, Stockwell TB, Walenz BP, Li K, Axelrod N, Busam DA, Strausberg RL, Venter JC. Genetic variation in an individual human exome. PLoS Genet. 2008 Aug 15;4(8):e1000160) reports that just under 50% of all nonsynonymous SNPs in HuRef are homozygous. However, they go on report that only about 15% are likely to affect protein function in a deleterious way and in this case, heterozygous nsSNPS are two times more likely than homozygotes to be predicted to affect protein activity. Similarly, rare nsSNPS are twice as likely as common nsSNPs to affect protein function. Thus, at the active site and other functionally important residues, protein-affecting SNPs should be selected against and should be less likely to be observed in homozygote form.

At your suggestion, we conducted a new PubMed search and were able to find an article identifying a homozygous mutation in the catalytic domain of the MMP2 gene in two sisters with Winchester syndrome (Rouzier C1, Vanatka R, Bannwarth S, Philip N, Coussement A, Paquis-Flucklinger V, Lambert JC. A novel homozygous MMP2 mutation in a family with Winchester syndrome. Clin Genet. 2006 Mar;69(3):271–6.) The long-term combination of (1) improved classification and annotation of variation data and (2) simple increase in available studies should allow us to find these cases and truly study the quantitative differences of heterozygous and homozygous states, ultimately helping to develop and refine possible detection assays.

The surprising finding, actually, seems to be that even under the stringent filtering used – conserved orthologs from yeast to human of enzymes in a conserved pathway – five of the six genes nominated for study are associated with described human genetic diseases, not simply non-viability. The alleles responsible for the genetic disorders seen in patients may encode enzymes that retain some residual function, probably more than an active site mutant retains, allowing for viability.

Rather than presenting a new method to find previously unrecognized human genetic disease, this paper forms a field test of the bioinformatics infrastructure one would need for such a pipeline in the future. If FBA is not yet available for human cells, the yeast model can stand in for a few validated human/yeast orthologs. When it turns out that zeroing activity of several genes causes no change, it suggests the FBA model needs repair. The authors describe making such repairs, on their way to completing their proof-of-principle for notion that a yeast metabolic model, and an infrastructure for data integration, could point the way to the prediction of diseases that could be caused by certain missense mutations.

Authors’ response: *Thank you very much for your detailed comments and review which helped us to focus our editing efforts and improve our arguments.*

### Reviewer 2: Dr. Igor B. Rogozin

The authors analyzed nonsynonymous single nucleotide variations (nsSNVs) in active site residues in human-yeast protein alignments. This is a promising approach. I do not see major methodological problems. The only problem that I see is a discussion about "loss of function via haploinsufficiency or dominant negative phenotypes". I understand that this is a complex problem. For example, the vast majority of disease-associated mutations in the RPE65 gene (Poliakov E, Gubin AN, Stearn O, Li Y, Campos MM, Gentleman S, Rogozin IB, Redmond TM. Origin and evolution of retinoid isomerization machinery in vertebrate visual cycle: hint from jawless vertebrates. PLoS One. 2012;7: e49975) are recessive mutations. However a dominant mutation was recently found in this gene. This and many other examples (including those described in the paper) suggest that the problem of dominant mutations is extremely complex. I am not sure that the fraction of such mutations is high although I am not aware of such estimate in active site residues.

Authors’ response: *Thank you for your positive comments regarding our approach and your general suggestions as well. We do realize the complexity of this issue and the necessity of exercising caution when applying models across organisms without knowing the full characterization of the deleterious outcome. As I mentioned in my response to Dr. Haft above, we have discussed the issue of zygosity, phenotype and loss-of-function both prior to the initial submission and since receipt of your responses. We do not want to ignore the modeling ramifications of differential ploidy, but we also don’t want to exclude candidate proteins from downstream quantification steps as it may be possible that future in vivo assays (developed as a product of the broader study) can still have some impact on heterozygous disease states even if in silico models cannot properly simulate them. We have carefully reread and edited these sections in response to your comments and we feel that we have clarified our reasons for our treatments of these issues.*

### Reviewer 3: Dr. I. King Jordan

This reviewer provided no comments for publication.

## Abbreviations

ALAD: Human gene encoding delta-aminolevulinic acid dehydratase; APIP: Human gene encoding methylthioribulose-1-phosphate dehydratase; ARS: Aminoacyl-tRNA synthetase; CID: PubChem Compound ID; CpG: Region of DNA where cytosine and guanine are separated by a single phosphate; EPP: Erythropoietic protoporphyria; FBA: Flux balance analysis; FECH: Human gene encoding ferrochelatase; HIVE: High-performance integrated virtual environment; MDE1: Yeast gene encoding methylthioribulose-1-phosphate dehydratase; MLASA: Myopathy with lactic acidosis and sideroblastic anaemia; MTA: Methylthioadenosine; nsSNV: Nonsynonymous single nucleotide variation; PPP: Pentose phosphate pathway; RKI1: Yeast gene encoding ribose 5-phosphate isomerase; RPI: Ribose 5-phosphate isomerase; RPIA: Human gene encoding RPI; SID: PubChem substance ID; SNP: Single nucleotide polymorphism; TyrRS: Mitochondrial tyrosyl-tRNA synthetase.

## Competing interests

The authors declare that they have no competing interests.

## Authors' contributions

RM, HD conceived and designed the analysis. HD carried out the manual analysis and identification of orthologs and drafted the manuscript. DW carried out all modeling experiments. YP performed reciprocal best BLAST hits method. DW and YP contributed text to relevant sections of manuscript. PDK, VS contributed analysis tools. All authors read and approved the final manuscript.

## Supplementary Material

Additional file 1: Table A1 SNVDis Results (duplicates removed). Results from initial SNVDis computation listing 934 SNPs found in annotated human active sites. **Table A2.** Recip. BLAST findings. Four column table: A – human uniprot accession; B – yeast best match to human protein from A in BLAST; C – human best match to yeast protein from B; D – yes if columns A and C match implying human and yeast proteins are reciprocal best matches and can therefore be considered for orthology. **Table A3.** Yeast 7.0 Outputs. Raw outputs from original and augmented Yeast 7.0 models. Click here for file

## References

[B1] Hartmann T Terao M Garattini E Teutloff C Alfaro JF Jones JP Leimkuhler S The impact of single nucleotide polymorphisms on human aldehyde oxidase Drug Metab Dispos 2012 9 856 864 10.1124/dmd.111.043828 22279051 PMC4738704

[B2] Li CY Yu Q Ye ZQ Sun Y He Q Li XM Zhang W Luo J Gu X Zheng X Wei L A nonsynonymous SNP in human cytosolic sialidase in a small Asian population results in reduced enzyme activity: potential link with severe adverse reactions to oseltamivir Cell Res 2007 9 357 362 10.1038/cr.2007.27 17426694

[B3] Aleem AM Wells L Jankun J Walther M Kuhn H Reinartz J Skrzypczak-Jankun E Human platelet 12-lipoxygenase: naturally occurring Q261/R261 variants and N544L mutant show altered activity but unaffected substrate binding and membrane association behavior Int J Mol Med 2009 9 759 764 19885615 10.3892/ijmm_00000289

[B4] Lee YJ Horie Y Wallace GR Choi YS Park JA Choi JY Song R Kang YM Kang SW Baek HJ Kitaichi N Meguro A Mizuki N Namba K Ishida S Kim J Niemczyk E Lee EY Song YW Ohno S Lee EB Genome-wide association study identifies GIMAP as a novel susceptibility locus for Behcet's disease Ann Rheum Dis 2013 9 1510 1516 10.1136/annrheumdis-2011-200288 23041938

[B5] Renz KG Cheetham BF Walkden-Brown SW Differentiation between pathogenic serotype 1 isolates of Marek's disease virus and the Rispens CVI988 vaccine in Australia using real-time PCR and high resolution melt curve analysis J Virol Methods 2013 9 144 152 10.1016/j.jviromet.2012.09.018 23041147

[B6] Antonopoulos AS Tousoulis D Antoniades C Miliou A Hatzis G Papageorgiou N Demosthenous M Tentolouris C Stefanadis C Genetic variability on adiponectin gene affects myocardial infarction risk: the role of endothelial dysfunction Int J Cardiol 2012 9 326 330 23041095 10.1016/j.ijcard.2012.09.053

[B7] Nemoto H Tate G Kishimoto K Saito M Shirahata A Umemoto T Matsubara T Goto T Mizukami H Kigawa G Mitsuya T Hibi K Heterozygous loss of NF2 is an early molecular alteration in well-differentiated papillary mesothelioma of the peritoneum Cancer Genet 2012 9 594 598 10.1016/j.cancergen.2012.08.005 23036697

[B8] Zeng BJ Wang ZH Ribeiro LA Leone P De Gasperi R Kim SJ Raghavan S Ong E Pastores GM Kolodny EH Identification and characterization of novel mutations of the aspartoacylase gene in non-Jewish patients with Canavan disease J Inherit Metab Dis 2002 9 557 570 10.1023/A:1022091223498 12638939

[B9] Zankl A Bonafe L Calcaterra V Di Rocco M Superti-Furga A Winchester syndrome caused by a homozygous mutation affecting the active site of matrix metalloproteinase 2 Clin Genet 2005 9 261 266 10.1111/j.1399-0004.2004.00402.x 15691365

[B10] Riballo E Doherty AJ Dai Y Stiff T Oettinger MA Jeggo PA Kysela B Cellular and biochemical impact of a mutation in DNA ligase IV conferring clinical radiosensitivity J Biol Chem 2001 9 31124 31132 10.1074/jbc.M103866200 11349135

[B11] Dingerdissen H Motwani M Karagiannis K Simonyan V Mazumder R Proteome-wide analysis of nonsynonymous single-nucleotide variations in active sites of human proteins FEBS J 2013 9 1542 1562 10.1111/febs.12155 23350563

[B12] Karagiannis K Simonyan V Mazumder R SNVDis: a proteome-wide analysis service for evaluating nsSNVs in protein functional sites and pathways Genomics Proteomics Bioinformatics 2013 9 122 126 10.1016/j.gpb.2012.10.003 23618375 PMC3807806

[B13] Wu TJ Shamsaddini A Pan Y Smith K Crichton DJ Simonyan V Mazumder R A framework for organizing cancer-related variations from existing databases, publications and NGS data using a High-performance Integrated Virtual Environment (HIVE) Database (Oxford) 2014 9 bau022 10.1093/database/bau022 24667251 PMC3965850

[B14] Aoki KF Kanehisa M Using the KEGG database resource Current protocols in bioinformatics/editoral board, Andreas D Baxevanis [et al] 2005 9 Unit 1 12 10.1002/0471250953.bi0112s11 18428742

[B15] Tatusov RL Fedorova ND Jackson JD Jacobs AR Kiryutin B Koonin EV Krylov DM Mazumder R Mekhedov SL Nikolskaya AN Rao BS Smirnov S Sverdlov AV Vasudevan S Wolf YI Yin JJ Natale DA The COG database: an updated version includes eukaryotes BMC Bioinforma 2003 9 41 10.1186/1471-2105-4-41 PMC222959 12969510

[B16] Noble D Modeling the heart–from genes to cells to the whole organ Science 2002 9 1678 1682 10.1126/science.1069881 11872832

[B17] Heavner BD Smallbone K Barker B Mendes P Walker LP Yeast 5 - an expanded reconstruction of the Saccharomyces cerevisiae metabolic network BMC Syst Biol 2012 9 55 10.1186/1752-0509-6-55 22663945 PMC3413506

[B18] Orth JD Thiele I Palsson BO What is flux balance analysis? Nat Biotech 2010 9 245 248 10.1038/nbt.1614 PMC3108565 20212490

[B19] Lakshmanan M Koh G Chung BK Lee DY Software applications for flux balance analysis Brief Bioinform 2012 9 108 122 23131418 10.1093/bib/bbs069

[B20] Toya Y Shimizu H Flux analysis and metabolomics for systematic metabolic engineering of microorganisms Biotechnol Adv 2013 9 818 826 10.1016/j.biotechadv.2013.05.002 23680193

[B21] Khandelwal RA Olivier BG Roling WF Teusink B Bruggeman FJ Community flux balance analysis for microbial consortia at balanced growth PLoS One 2013 9 e64567 10.1371/journal.pone.0064567 23741341 PMC3669319

[B22] Beg QK Zampieri M Klitgord N Collins SB Altafini C Serres MH Segre D Detection of transcriptional triggers in the dynamics of microbial growth: application to the respiratorily versatile bacterium Shewanella oneidensis Nucleic Acids Res 2012 9 7132 7149 10.1093/nar/gks467 22638572 PMC3424579

[B23] Varma A Boesch BW Palsson BO Stoichiometric interpretation of Escherichia coli glucose catabolism under various oxygenation rates Appl Environ Microbiol 1993 9 2465 2473 8368835 10.1128/aem.59.8.2465-2473.1993 PMC182307

[B24] Lorenz A Osman F Sun W Nandi S Steinacher R Whitby MC The fission yeast FANCM ortholog directs non-crossover recombination during meiosis Science 2012 9 1585 1588 10.1126/science.1220111 22723423 PMC3399777

[B25] Ewald JC Matt T Zamboni N The integrated response of primary metabolites to gene deletions and the environment Mol BioSyst 2013 9 440 446 10.1039/c2mb25423a 23340584

[B26] Oliveira AP Ludwig C Picotti P Kogadeeva M Aebersold R Sauer U Regulation of yeast central metabolism by enzyme phosphorylation Mol Syst Biol 2012 9 623 23149688 10.1038/msb.2012.55 PMC3531909

[B27] Cooper SJ Finney GL Brown SL Nelson SK Hesselberth J MacCoss MJ Fields S High-throughput profiling of amino acids in strains of the Saccharomyces cerevisiae deletion collection Genome Res 2010 9 1288 1296 10.1101/gr.105825.110 20610602 PMC2928507

[B28] Consortium U Reorganizing the protein space at the universal protein resource (UniProt) Nucleic Acids Res 2012 9 D71 D75 22102590 10.1093/nar/gkr981 PMC3245120

[B29] Sherry ST Ward MH Kholodov M Baker J Phan L Smigielski EM Sirotkin K dbSNP: the NCBI database of genetic variation Nucleic Acids Res 2001 9 308 311 10.1093/nar/29.1.308 11125122 PMC29783

[B30] Marchler-Bauer A Lu S Anderson JB Chitsaz F Derbyshire MK DeWeese-Scott C Fong JH Geer LY Geer RC Gonzales NR Gwadz M Hurwitz DI Jackson JD Ke Z Lanczycki CJ Lu F Marchler GH Mullokandov M Omelchenko MV Robertson CL Song JS Thanki N Yamashita RA Zhang D Zhang N Zheng C Bryant SH CDD: a conserved domain database for the functional annotation of proteins Nucleic Acids Res 2011 9 D225 D229 10.1093/nar/gkq1189 21109532 PMC3013737

[B31] Ng PC Levy S Huang J Stockwell TB Walenz BP Li K Axelrod N Busam DA Strausberg RL Venter JC Genetic variation in an individual human exome PLoS Genet 2008 9 e1000160 10.1371/journal.pgen.1000160 18704161 PMC2493042

[B32] Rouzier C Vanatka R Bannwarth S Philip N Coussement A Paquis-Flucklinger V Lambert JC A novel homozygous MMP2 mutation in a family with Winchester syndrome Clin Genet 2006 9 271 276 10.1111/j.1399-0004.2006.00584.x 16542393

[B33] Venkatesan RN Treuting PM Fuller ED Goldsby RE Norwood TH Gooley TA Ladiges WC Preston BD Loeb LA Mutation at the polymerase active site of mouse DNA polymerase delta increases genomic instability and accelerates tumorigenesis Mol Cell Biol 2007 9 7669 7682 10.1128/MCB.00002-07 17785453 PMC2169052

[B34] Ponamarev MV Longley MJ Nguyen D Kunkel TA Copeland WC Active site mutation in DNA polymerase gamma associated with progressive external ophthalmoplegia causes error-prone DNA synthesis J Biol Chem 2002 9 15225 15228 10.1074/jbc.C200100200 11897778

[B35] Lawson AJ Walker EA Lavery GG Bujalska IJ Hughes B Arlt W Stewart PM Ride JP Cortisone-reductase deficiency associated with heterozygous mutations in 11beta-hydroxysteroid dehydrogenase type 1 Proc Natl Acad Sci U S A 2011 9 4111 4116 10.1073/pnas.1014934108 21325058 PMC3054023

[B36] Altschul SF Gish W Miller W Myers EW Lipman DJ Basic local alignment search tool J Mol Biol 1990 9 403 410 10.1016/S0022-2836(05)80360-2 2231712

[B37] Lim YH Charette JM Baserga SJ Assembling a protein-protein interaction map of the SSU processome from existing datasets PLoS One 2011 9 e17701 10.1371/journal.pone.0017701 21423703 PMC3053386

[B38] Singh R Xu J Berger B Global alignment of multiple protein interaction networks with application to functional orthology detection Proc Natl Acad Sci U S A 2008 9 12763 12768 10.1073/pnas.0806627105 18725631 PMC2522262

[B39] Orchard S Ammari M Aranda B Breuza L Briganti L Broackes-Carter F Campbell NH Chavali G Chen C del Toro N Duesbury M Dumousseau M Galeota E Hinz U Iannuccelli M Jagannathan S Jimenez R Khadake J Lagreid A Licata L Lovering RC Meldal B Melidoni AN Milagros M Peluso D Perfetto L Porras P Raghunath A Ricard-Blum S Roechert B The MIntAct project–IntAct as a common curation platform for 11 molecular interaction databases Nucleic Acids Res 2014 9 D358 D363 10.1093/nar/gkt1115 24234451 PMC3965093

[B40] Chatr-Aryamontri A Breitkreutz BJ Heinicke S Boucher L Winter A Stark C Nixon J Ramage L Kolas N O'Donnell L Reguly T Breitkreutz A Sellam A Chen D Chang C Rust J Livstone M Oughtred R Dolinski K Tyers M The BioGRID interaction database: 2013 update Nucleic Acids Res 2013 9 D816 D823 10.1093/nar/gks1158 23203989 PMC3531226

[B41] Online Mendelian Inheritance in Man, OMIM® [http://omim.org/] 17642958

[B42] Wamelink MM Struys EA Jakobs C The biochemistry, metabolism and inherited defects of the pentose phosphate pathway: a review J Inherit Metab Dis 2008 9 703 717 10.1007/s10545-008-1015-6 18987987

[B43] Berg JM Tymoczko JL Stryer L The Calvin Cycle and the Pentose Phosphate Pathway Biochemistry 2007 6 New York, NY: W.H. Freeman and Company 565 591

[B44] Kim JH Kang HS Kim TW Kim SJ Differential methylation hybridization profiling identifies involvement of STAT1-mediated pathways in breast cancer Int J Oncol 2011 9 955 963 21674123 10.3892/ijo.2011.1075

[B45] Huck JH Verhoeven NM Struys EA Salomons GS Jakobs C van der Knaap MS Ribose-5-phosphate isomerase deficiency: new inborn error in the pentose phosphate pathway associated with a slowly progressive leukoencephalopathy Am J Hum Genet 2004 9 745 751 10.1086/383204 14988808 PMC1181951

[B46] Stenson PD Ball EV Mort M Phillips AD Shaw K Cooper DN The human gene mutation database (HGMD) and its exploitation in the fields of personalized genomics and molecular evolution Curr Protoc Bioinformatics 2012 9 Unit1 13 22948725 10.1002/0471250953.bi0113s39

[B47] Apel TW Scherer A Adachi T Auch D Ayane M Reth M The ribose 5-phosphate isomerase-encoding gene is located immediately downstream from that encoding murine immunoglobulin kappa Gene 1995 9 191 197 10.1016/0378-1119(94)00901-4 7758956

[B48] Kondo H Nakamura Y Dong YX Nikawa J Sueda S Pyridoxine biosynthesis in yeast: participation of ribose 5-phosphate ketol-isomerase Biochem J 2004 9 65 70 10.1042/BJ20031268 14690456 PMC1224052

[B49] Lawrence SH Ramirez UD Selwood T Stith L Jaffe EK Allosteric inhibition of human porphobilinogen synthase J Biol Chem 2009 9 35807 35817 10.1074/jbc.M109.026294 19812033 PMC2791010

[B50] Bardag-Gorce F French SW Delta-aminolevulinic dehydratase is a proteasome interacting protein Exp Mol Pathol 2011 9 485 489 10.1016/j.yexmp.2011.05.003 21640720 PMC3185203

[B51] Inoue R Akagi R Co-synthesis of human delta-aminolevulinate dehydratase (ALAD) mutants with the wild-type enzyme in cell-free system-critical importance of conformation on enzyme activity J Clin Biochem Nutr 2008 9 143 153 10.3164/jcbn.2008035 19015748 PMC2581755

[B52] Lawrence SH Selwood T Jaffe EK Diverse clinical compounds alter the quaternary structure and inhibit the activity of an essential enzyme ChemMedChem 2011 9 1067 1073 10.1002/cmdc.201100009 21506274 PMC3236527

[B53] Jaffe EK Morpheeins - a new pathway for allosteric drug discovery Open Conf Proc J 2010 9 1 6 21643557 10.2174/2210289201001010001 PMC3107518

[B54] Gollub EG Liu KP Dayan J Adlersberg M Sprinson DB Yeast mutants deficient in heme biosynthesis and a heme mutant additionally blocked in cyclization of 2,3-oxidosqualene J Biol Chem 1977 9 2846 2854 323256

[B55] Jaffe EK Martins J Li J Kervinen J Dunbrack RL Jr The molecular mechanism of lead inhibition of human porphobilinogen synthase J Biol Chem 2001 9 1531 1537 10.1074/jbc.M007663200 11032836

[B56] Erskine PT Newbold R Brindley AA Wood SP Shoolingin-Jordan PM Warren MJ Cooper JB The x-ray structure of yeast 5-aminolaevulinic acid dehydratase complexed with substrate and three inhibitors J Mol Biol 2001 9 133 141 10.1006/jmbi.2001.4947 11545591

[B57] Akagi R Shimizu R Furuyama K Doss MO Sassa S Novel molecular defects of the delta-aminolevulinate dehydratase gene in a patient with inherited acute hepatic porphyria Hepatology 2000 9 704 708 10.1002/hep.510310321 10706561

[B58] Jaffe EK Stith L ALAD porphyria is a conformational disease Am J Hum Genet 2007 9 329 337 10.1086/511444 17236137 PMC1785348

[B59] Medlock A Swartz L Dailey TA Dailey HA Lanzilotta WN Substrate interactions with human ferrochelatase Proc Natl Acad Sci U S A 2007 9 1789 1793 10.1073/pnas.0606144104 17261801 PMC1794275

[B60] Lamoril J Boulechfar S de Verneuil H Grandchamp B Nordmann Y Deybach JC Human erythropoietic protoporphyria: two point mutations in the ferrochelatase gene Biochem Biophys Res Commun 1991 9 594 599 10.1016/0006-291X(91)91231-Z 1755842

[B61] Aurizi C Schneider-Yin X Sorge F Macri A Minder EI Biolcati G Heterogeneity of mutations in the ferrochelatase gene in Italian patients with erythropoietic protoporphyria Mol Genet Metab 2007 9 402 407 10.1016/j.ymgme.2006.10.012 17196862

[B62] Schneider-Yin X Gouya L Dorsey M Rufenacht U Deybach JC Ferreira GC Mutations in the iron-sulfur cluster ligands of the human ferrochelatase lead to erythropoietic protoporphyria Blood 2000 9 1545 1549 10942404

[B63] Labbe-Bois R The ferrochelatase from Saccharomyces cerevisiae. Sequence, disruption, and expression of its structural gene HEM15 J Biol Chem 1990 9 7278 7283 2185242

[B64] Yao P Fox PL Aminoacyl-tRNA synthetases in medicine and disease EMBO Mol Med 2013 9 332 343 10.1002/emmm.201100626 23427196 PMC3598075

[B65] Bonnefond L Frugier M Giege R Rudinger-Thirion J Human mitochondrial TyrRS disobeys the tyrosine identity rules RNA 2005 9 558 562 10.1261/rna.7246805 15840810 PMC1370743

[B66] Riley LG Cooper S Hickey P Rudinger-Thirion J McKenzie M Compton A Lim SC Thorburn D Ryan MT Giege R Bahlo M Christodoulou J Mutation of the mitochondrial tyrosyl-tRNA synthetase gene, YARS2, causes myopathy, lactic acidosis, and sideroblastic anemia–MLASA syndrome Am J Hum Genet 2010 9 52 59 10.1016/j.ajhg.2010.06.001 20598274 PMC2896778

[B67] Wakasugi K Schimmel P Two distinct cytokines released from a human aminoacyl-tRNA synthetase Science 1999 9 147 151 10.1126/science.284.5411.147 10102815

[B68] Wakasugi K Slike BM Hood J Ewalt KL Cheresh DA Schimmel P Induction of angiogenesis by a fragment of human tyrosyl-tRNA synthetase J Biol Chem 2002 9 20124 20126 10.1074/jbc.C200126200 11956181

[B69] Bonnefond L Fender A Rudinger-Thirion J Giege R Florentz C Sissler M Toward the full set of human mitochondrial aminoacyl-tRNA synthetases: characterization of AspRS and TyrRS Biochemistry 2005 9 4805 4816 10.1021/bi047527z 15779907

[B70] Bonnefond L Frugier M Touze E Lorber B Florentz C Giege R Sauter C Rudinger-Thirion J Crystal structure of human mitochondrial tyrosyl-tRNA synthetase reveals common and idiosyncratic features Structure 2007 9 1505 1516 10.1016/j.str.2007.09.018 17997975

[B71] Albers E Metabolic characteristics and importance of the universal methionine salvage pathway recycling methionine from 5'-methylthioadenosine IUBMB Life 2009 9 1132 1142 10.1002/iub.278 19946895

[B72] Mary C Duek P Salleron L Tienz P Bumann D Bairoch A Lane L Functional identification of APIP as human mtnB, a key enzyme in the methionine salvage pathway PLoS One 2012 9 e52877 10.1371/journal.pone.0052877 23285211 PMC3532061

[B73] Cho DH Hong YM Lee HJ Woo HN Pyo JO Mak TW Jung YK Induced inhibition of ischemic/hypoxic injury by APIP, a novel Apaf-1-interacting protein J Biol Chem 2004 9 39942 39950 10.1074/jbc.M405747200 15262985

[B74] Ko DC Gamazon ER Shukla KP Pfuetzner RA Whittington D Holden TD Brittnacher MJ Fong C Radey M Ogohara C Stark AL Akey JM Dolan ME Wurfel MM Miller SI Functional genetic screen of human diversity reveals that a methionine salvage enzyme regulates inflammatory cell death Proc Natl Acad Sci U S A 2012 9 E2343 E2352 10.1073/pnas.1206701109 22837397 PMC3435171

[B75] Karp PD Ouzounis CA Moore-Kochlacs C Goldovsky L Kaipa P Ahren D Tsoka S Darzentas N Kunin V Lopez-Bigas N Expansion of the BioCyc collection of pathway/genome databases to 160 genomes Nucleic Acids Res 2005 9 6083 6089 10.1093/nar/gki892 16246909 PMC1266070

[B76] Pirkov I Norbeck J Gustafsson L Albers E A complete inventory of all enzymes in the eukaryotic methionine salvage pathway FEBS J 2008 9 4111 4120 10.1111/j.1742-4658.2008.06552.x 18625006

[B77] Forbes SA Bindal N Bamford S Cole C Kok CY Beare D Jia M Shepherd R Leung K Menzies A Teague JW Campbell PJ Stratton MR Futreal PA COSMIC: mining complete cancer genomes in the catalogue of somatic mutations in cancer Nucleic Acids Res 2011 9 D945 D950 10.1093/nar/gkq929 20952405 PMC3013785

[B78] Abaan OD Polley EC Davis SR Zhu YJ Bilke S Walker RL Pineda M Gindin Y Jiang Y Reinhold WC Holbeck SL Simon RM Doroshow JH Pommier Y Meltzer PS The exomes of the NCI-60 panel: a genomic resource for cancer biology and systems pharmacology Cancer Res 2013 9 4372 4382 10.1158/0008-5472.CAN-12-3342 23856246 PMC4893961

[B79] Tanabe M Kanehisa M Using the KEGG database resource Current protocols in bioinformatics/editoral board, Andreas D Baxevanis [et al] 2012 9 Unit1 12 10.1002/0471250953.bi0112s38 22700311

[B80] Mi H Guo N Kejariwal A Thomas PD PANTHER version 6: protein sequence and function evolution data with expanded representation of biological pathways Nucleic Acids Res 2007 9 D247 D252 10.1093/nar/gkl869 17130144 PMC1716723

[B81] Bolton E Wang Y Thiessen PA Bryant SH PubChem: Integrated Platform of Small Molecules and Biological Activities. Chapter 12 Annual Reports in Computational Chemistry Volume 4, American Chemical Society; Washington, DC, 2008 Apr. http://oldwww.acscomp.org/Publications/ARCC/volume4/chapter12.html

[B82] Altschul SF Madden TL Schaffer AA Zhang J Zhang Z Miller W Lipman DJ Gapped BLAST and PSI-BLAST: a new generation of protein database search programs Nucleic Acids Res 1997 9 3389 3402 10.1093/nar/25.17.3389 9254694 PMC146917

[B83] Cherry JM Hong EL Amundsen C Balakrishnan R Binkley G Chan ET Christie KR Costanzo MC Dwight SS Engel SR Fisk DG Hirschman JE Hitz BC Karra K Krieger CJ Miyasato SR Nash RS Park J Skrzypek MS Simison M Weng S Wong ED Saccharomyces genome database: the genomics resource of budding yeast Nucleic Acids Res 2012 9 D700 D705 10.1093/nar/gkr1029 22110037 PMC3245034

